# Neuroinflammation, Energy and Sphingolipid Metabolism Biomarkers Are Revealed by Metabolic Modeling of Autistic Brains

**DOI:** 10.3390/biomedicines11020583

**Published:** 2023-02-16

**Authors:** Elif Esvap, Kutlu O. Ulgen

**Affiliations:** Department of Chemical Engineering, Bogazici University, Istanbul 34342, Turkey

**Keywords:** autism spectrum disorder, genome-scale metabolic modeling, neuroinflammation, oxidative stress, mitochondrial dysfunction, sphingolipid

## Abstract

Autism spectrum disorders (ASD) are a heterogeneous group of neurodevelopmental disorders generally characterized by repetitive behaviors and difficulties in communication and social behavior. Despite its heterogeneous nature, several metabolic dysregulations are prevalent in individuals with ASD. This work aims to understand ASD brain metabolism by constructing an ASD-specific prefrontal cortex genome-scale metabolic model (GEM) using transcriptomics data to decipher novel neuroinflammatory biomarkers. The healthy and ASD-specific models are compared via uniform sampling to identify ASD-exclusive metabolic features. Noticeably, the results of our simulations and those found in the literature are comparable, supporting the accuracy of our reconstructed ASD model. We identified that several oxidative stress, mitochondrial dysfunction, and inflammatory markers are elevated in ASD. While oxidative phosphorylation fluxes were similar for healthy and ASD-specific models, and the fluxes through the pathway were nearly undisturbed, the tricarboxylic acid (TCA) fluxes indicated disruptions in the pathway. Similarly, the secretions of mitochondrial dysfunction markers such as pyruvate are found to be higher, as well as the activities of oxidative stress marker enzymes like alanine and aspartate aminotransferases (ALT and AST) and glutathione-disulfide reductase (GSR). We also detected abnormalities in the sphingolipid metabolism, which has been implicated in many inflammatory and immune processes, but its relationship with ASD has not been thoroughly explored in the existing literature. We suggest that important sphingolipid metabolites, such as sphingosine-1-phosphate (S1P), ceramide, and glucosylceramide, may be promising biomarkers for the diagnosis of ASD and provide an opportunity for the adoption of early intervention for young children.

## 1. Introduction

Autism spectrum disorder (ASD) is a neurodevelopmental disorder that shows up in early childhood and is a lifelong condition. Although the heterogeneous nature of this disorder creates difficulties when diagnosing people, most ASD patients lack verbal and nonverbal communication skills, insist on the same routine, and show restricted and repetitive behaviors [1]. The prevalence of ASD among children varies worldwide, ranging from 0.01% to 4.36%, with higher prevalence values in high-income countries. Methodological, cultural, and environmental variables, as well as access to mental healthcare and research funding, may all play a role in variations in prevalence values. Nevertheless, two conclusions were prominently similar in all reports: the prevalence of ASD increases year by year, and males are, on average, four times more susceptible than females [2].

A number of studies emphasized that various brain parts, such as the frontal lobes, amygdala, and cerebellum, may show structural and functional differences in ASD and can take part in the pathology of the disorder [3]. For instance, the cortical thickness of the frontal cortex was found to be increased in the frontal cortex but decreased in the temporal cortex of the ASD group [4]. Also, people with ASD showed functional brain abnormalities, such as decreased activity in the left dorsolateral prefrontal cortex [5]. The prefrontal cortex, which covers the front part of the frontal lobe, is responsible for cognitive, language, social, and emotional functions, and ASD patients are known to have verbal and social difficulties [6]. Compared with neurotypical controls, the number of neurons and size of the prefrontal cortex are greater in ASD children [7]. In addition, microglial activation and microglial somal volume are increased in the dorsolateral prefrontal cortex of ASD children [8]. Transcriptomics, proteomics, and metabolomics studies have also shown significant differences between the frontal cortexes of ASD and neurotypical controls [9,10].

Several central nervous system-based cellular and metabolic dysregulations, such as inflammation, oxidative stress, and mitochondrial dysfunction have been implicated in various neurodegenerative, neuropsychiatric, and neurodevelopmental diseases such as Alzheimer’s disease, schizophrenia, bipolar disease, depression, and ASD [11]. Specifically, pro-inflammatory cytokines, such as IL-1β, IL-6, IL-17, and TNF-α, and pro-inflammatory metabolites, such as neopterin and prostaglandins, were higher in the bodily fluids and brains of ASD individuals, while the levels of anti-inflammatory markers (i.e., choline, myo-inositol) were shown to be lower [12,13,14]. Similarly, oxidative stress signals, including drops in the reduced glutathione (GSH) and GSH/GSSG redox ratio [15] and mitochondrial dysfunction markers, such as elevated levels of pyruvate, lactate, ammonia, alanine aminotransferase (ALT), and aspartate aminotransferase (AST), have been identified in ASD patients [16].

Although genome-scale metabolic models (GEM) are comparatively new, they are very useful systems biology tools that can provide a holistic model for the whole metabolism and can also be used to decipher the relationship between genotype and phenotype and predict the effects of environmental changes on the metabolism. GEMs have been used for many applications, such as cell factory design for the production of chemicals and prediction of microbial community interactions [17]. Additionally, GEMs have also been utilized for the detection of metabolic biomarkers in human diseases, such as cancer and neuropsychiatric disorders [18,19].

Despite the fact that ASD is a prevalent disorder, the underlying mechanisms behind its pathogenesis are still unknown. The current work aims to investigate to which extent several cellular processes, including neuroinflammation and oxidation stress, in the prefrontal cortex of ASD children are perturbed at the genome-scale. Thus, ASD-specific and neurotypical prefrontal cortex genome-scale metabolic models have been constructed using transcriptomics data to better understand the ASD brain metabolism and consequently to decipher novel biomarkers for ASD. To uncover the ASD-specific metabolic traits, the healthy and ASD-specific models were compared, and the increased neuroinflammatory and oxidative stress-related markers in the ASD model identified. Understanding the metabolic reprogramming in autistic brains not only sheds light on the molecular mechanisms that lead to ASD, but it may also help to direct early intervention strategies.

## 2. Materials and Methods

### 2.1. ASD-Specific Prefrontal Cortex GEM Construction

The postmortem prefrontal cortex transcriptome data for autistic people and neurotypical controls aged between 2 and 56 were acquired from the Gene Expression Omnibus database with the ID GSE28475 [20,21]. The samples were collected from the superior frontal gyrus of the dorsolateral prefrontal cortex or the middle frontal gyrus, if the former was unavailable. For the current work, only the samples acquired by DASL-based assays were included in the analysis to keep the data consistent. Since the gene-expression levels are dependent on age, as shown by another study by the same group [22], we chose to use the median expressions for the samples aged between 2 and 14.

**Figure 1:**
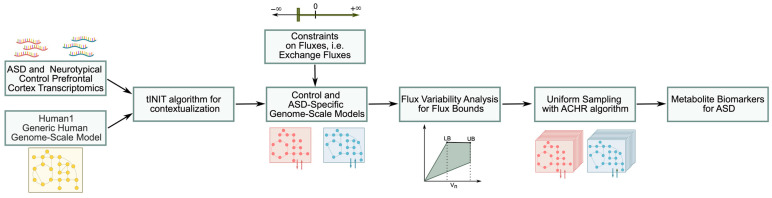
Flowchart for the biomarker investigation.

A flowchart of the methodology used in this work can be seen in Figure 1. tINIT [23] was chosen as the contextualizing algorithm, and Human1 [24] was selected as the reference model, since it unifies all the information from the other three generic human metabolic models, namely HMR 2.0 [25], iHsa [26] and Recon3D [27], and has more metabolites and reactions present than the other three models. The decision for threshold selection in the tINIT algorithm was made according to the distribution of expression values. The distribution of the quantile normalized gene expression values is bimodal, with a local minimum between 9 and 9.25. We selected 9.25 as the threshold since lower values tend to increase the number of reactions and metabolites in the model while decreasing the specificity of the model (Figure S1).

The RAVEN toolbox [28] was used during GEM contextualization, and the simulations were performed using the COBRA toolbox [29]. All related operations were conducted in MATLAB R2017b, and Gurobi optimization software (version 8.1.1) [30] was used as the linear programming solver. In both the ASD-specific and neurotypical models, we checked if the pruned models contained all the reactions regarding the glutamine–glutamate–GABA cycle, and if absent, we added them manually. Additionally, if the exchange and transport reactions for these and other essential metabolites are missing from the pruned models but not from the reference Human1, they were included in the models.

### 2.2. GEM Constraints

For the ASD and control models, separate reaction bounds were set if the information was available. The glucose uptake rate was set to the cerebral metabolic rate (CMR) of glucose in the frontal cortexes of the ASD and neurotypical children [31]. The maximum oxygen uptake rates were set according to the metabolic ratios of CMRO_2_/CMRglu for the young ASD patients and controls [32], and the minimum oxygen uptake rates were set to the minimum of CMRglu in the mild asphyxia infants [33]. The energy expenditure is estimated to be nearly 10 µmol ATP/(g min) for non-signaling cellular activities and 30 µmol ATP/(g min) for signaling-related processes, and these were set as lower (non-signaling) and upper (signaling + non-signaling) boundaries for the non-growth-associated ATP maintenance reaction [34]. The maximum reaction rates for the glutaminase [35], glutamine synthetase [36], and glutamate carboxylase [37] reactions were set as the greater V_max_ value between astrocytes and neurons. For the exchange reactions of other essential nutrients, such as amino acids, the cerebrospinal fluid (CSF) concentrations were converted to flux units using CSF flow rates, and these were assumed to be equal to the maximum uptake rates for the corresponding reactions [38].

### 2.3. Model Simulations

Most steady-state analysis methods, such as flux balance analysis (FBA), require a predefined objective function for the optimization of the problem. For organisms like bacteria, the selection of the objective function is mostly straightforward; they aim to maximize their growth and proliferate. However, for human cells, especially for brain cortex cells, maximization of the growth function is not applicable. Thus, we maximized the reaction rates for the glutaminase, glutamine synthetase, and glutamate carboxylase reactions to provide the replenishment of the glutamine in the neurotransmitter pool through the glutamine–glutamate–GABA cycle. Uniform sampling was used in order to obtain the distribution of attainable flux values rather than one of the optima obtained by the FBA. The lower and upper bounds of the reactions were set to the minimum and maximum flux values obtained through flux variability analysis (FVA) with a loopless solution option by maximizing the glutamine–glutamate–GABA cycle, if possible. A total of 10,000 samples were collected using the artificial centering hit-and-run (ACHR) algorithm within the COBRA Toolbox.

### 2.4. Statistical Analysis

All statistical analyses and data visualizations were performed in R software version 3.6.3. Due to the large dataset, effect size estimations were reported instead of the significance of the difference between group means/medians. The effect sizes between samples were determined using Cliff’s d using the effsize R package [39]. The Cliff’s d values were computed using an unbiased estimate formula, non-normal distribution assumption, and 95% confidence interval levels.

## 3. Results and Discussion

### 3.1. Model, Constraint, and Simulation Features

The selection of the flux constraints for model reactions is a significant step in genome-scale modeling. The phenotype of an organism or cell is determined by its environment and thermodynamic limitations, as well as by its genotype. Thus, appropriate constraints should be set for the simulations to obtain a realistic set of solutions. Since the metabolisms of children and adults are dissimilar, the search for the constraints was concentrated on experimental data for children aged 2–14, but if the information was not available, the values obtained from the adult ASD and control cases were used.

The extracellular fluid of the brain and spinal cord consists of blood plasma, cerebrospinal fluid (CSF), and interstitial fluid (ISF). The brain tissue is surrounded by ISF, while the CSF fills the brain ventricles and subarachnoid space. The compositions of ISF and CSF are very similar, although they are significantly different from the composition of blood since the blood–brain barrier is a highly selective barrier that allows the passage of small non-polar molecules and lipophilic molecules and blocks the passage of molecules such as neurotransmitters out of the brain [40]. The compositions of these extracellular fluids are substantial for the brain’s functioning. One study showed that the concentrations of several amino acids in CSF, such as glycine, aspartate, and arginine, are significantly different in ASD children compared to other patients with neurological problems [38]. The number of significantly different amino acid concentrations might be even higher if ASD and neurotypical children are compared; however, since it is not ethical to perform a lumbar puncture on healthy children to obtain CSF, no studies have investigated this difference. Yet, the presented information may give insight into the metabolism differences between ASD patients and others. Thus, the maximum amino acid uptake rates are set to their concentrations in CSF after the necessary conversions are done.

The glucose utilization rates were also determined to be not significantly different between ASD and control children aged between 2 and 18 [31]. The metabolic ratio of the cerebral metabolic rate of oxygen to that of glucose has not been reported for ASD; thus, in this study, the value for young adults was used to determine the maximum uptake rate of the oxygen.

**Figure 2:**
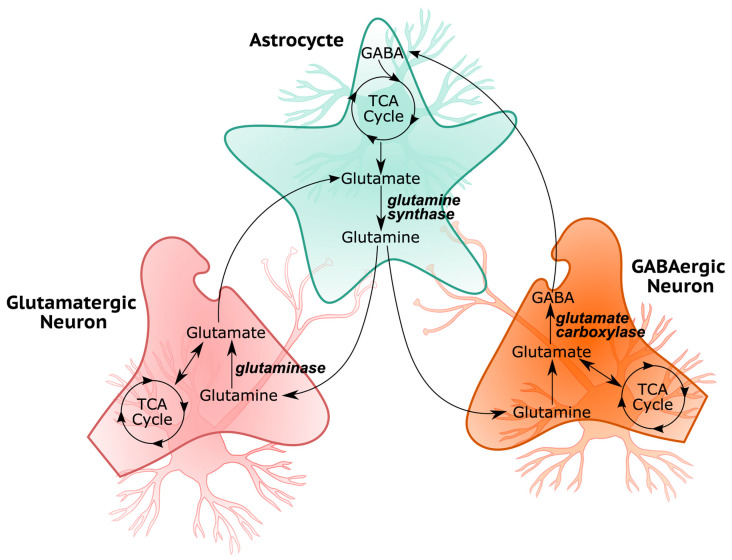
Simplified illustration of glutamine–glutamate–GABA cycle in the brain cortex.

The human prefrontal cortex mostly consists of astrocytes, glutamatergic excitatory pyramidal neurons, and GABAergic inhibitory interneurons. A simplified illustration of the exchanges between the astrocytes and neurons can be seen in Figure 2. Glutamate and gamma-aminobutyric acid (GABA) are two essential neurotransmitters: the former acts as excitatory, and the latter acts as an inhibitor. Neurons lack the ability to produce glutamate de novo from glucose since pyruvate carboxylase is not present in neurons [41]. Glutamate is converted into glutamine in astrocytes and released into extracellular space to later be used as precursors for glutamate and GABA in glutamatergic and GABAergic neurons, respectively. Since the neurotransmitter exchange between these cells is crucial for the functioning of the prefrontal cortex, the objective functions for both models are here selected as the maximization of the glutaminase, glutamine synthetase, and glutamate carboxylase reactions for the flux variability analysis, which is later used as the flux bounds of the model reactions for the uniform sampling analysis. Although the ACHR sampling algorithm used here randomly picks an objective function among the model’s reactions for each sampling, constraining all reactions to the FVA results provided non-zero flux through the glutamine–glutamate–GABA cycle for each sample.

### 3.2. Cellular Respiration and Energy Metabolism Is Disturbed in ASD

Mitochondria is the main ATP generation unit of the cells through oxidative phosphorylation and is also responsible for the regulation of reactive oxygen species (ROS). Although only 5% of ASD children are diagnosed with mitochondrial disease, up to 80% of ASD children show signs of mitochondrial dysfunction [16]. Mitochondrial dysfunction biomarkers were found to be elevated in the blood, plasma, and urine samples of individuals with ASD [42]. On the other hand, a large amount of cellular ROS is produced in mitochondria as a byproduct of electron transport chain (ETC) activity.

The brain only constitutes 2% of a human’s body weight, yet it accounts for nearly 20% of the body’s required daily energy. Glucose is the primary energy source for brain cells, and glycolysis, the tricarboxylic acid (TCA) cycle and oxidative phosphorylation are the mechanisms for producing ATP by breaking glucose. Theoretically, a maximum of 36 ATPs can be produced per consumed glucose molecule, two produced by glycolysis, two by the TCA cycle, and 32 by oxidative phosphorylation. Several studies detected that the genes that play a role in oxidative phosphorylation are downregulated in the brains of autistic patients [43,44].

**Figure 3:**
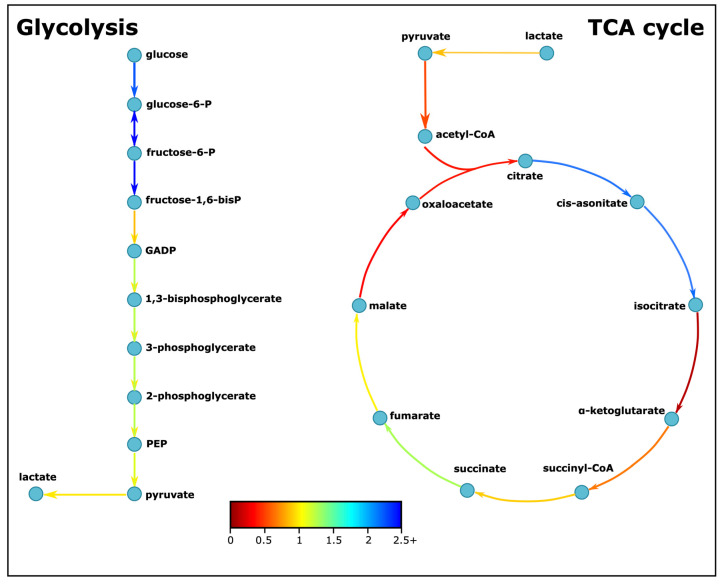
Simplified glycolysis and tricarboxylic acid cycle pathways. The colors indicate the ratio of average fluxes of ASD to control model. GADP: Glyceraldehyde-3-P, PEP: Phosphoenolpyruvate.

**Figure 4:**
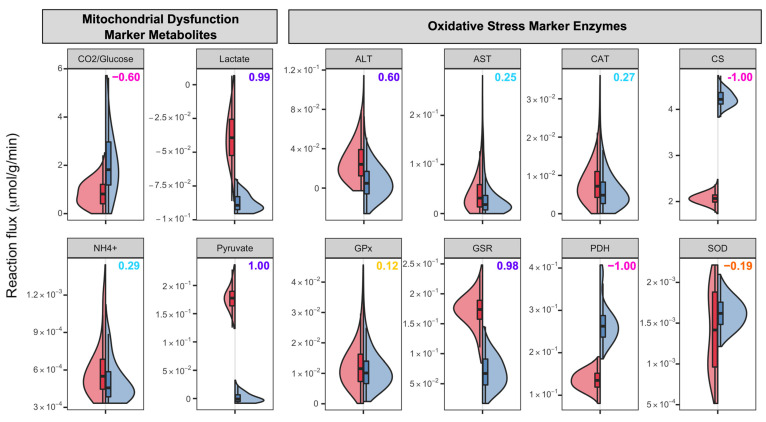
Reaction fluxes for several mitochondrial dysfunction and oxidative stress markers in µmol/(g min) acquired through the uniform sampling of ASD-specific and control models. For the metabolites and enzymes, the reaction fluxes indicate the secretion rate of the metabolite or the sum of the reaction fluxes catalyzed by the enzyme in all compartments, respectively. The numbers of the upper right corner of each graph denote the Cliff’s d values. The color codes for Cliff’s d values can be seen in Figure 5. ALT: Alanine aminotransferase, AST: Aspartate aminotransferase, CAT: Catalase, CS: Citrate synthase, GPx: Glutathione peroxidase, GSR: Glutathione-disulfide reductase, PDH: Pyruvate dehydrogenase, SOD: Superoxide dismutase.

In our genome-scale metabolic brain model, the attainable flux distributions for the aerobic respiration-related pathways showed that the glycolysis pathway is more active in the ASD model (Table 1 and Figure 3). In parallel with these results, one study conducted on lymphoblastoid cell lines (LCLs) from ASD patients, their siblings, and controls demonstrated that the glycolytic capacities of ASD LCLs were 19% and 12% greater than their siblings and unrelated controls, respectively [45]. While the ratios of the average of these calculated fluxes are similar through glycolysis, the ratios are variable through the TCA cycle. The fluxes converting citrate, cis-aconitate, and succinate are faster, and fluxes converting isocitrate, α-ketoglutarate, malate, and oxaloacetate are predicted to be at least 40% slower in the ASD model. The inconsistent flux levels observed in the TCA cycle of the ASD model compared to the control model suggest that metabolites downstream of isocitrate are differentially produced in ASD and that there is a disruption in the TCA cycle. One may speculate that two of these consequent downstream enzymes, isocitrate dehydrogenase (IDH) and α-ketoglutarate dehydrogenase (α-KGDH), are activated and regulated by calcium ions (Ca^2+^) in the mitochondrial matrix [46], and the deficiency of Ca^2+^ may create disruptions in the mitochondrial metabolism. Indeed, genetic variants of voltage-gated calcium channels (VGCCs), transmembrane proteins mediating the Ca^2+^ flux to neurons, were detected in ASD and speculated to be a part of the pathophysiology of ASD [47]. Alterations in TCA metabolite levels were also detected in other studies [48,49,50]. The changes in the TCA metabolites may indicate that the TCA cycle is a common pathway impacted in ASD. Consequently, as our computational findings point out, the increase in glycolysis rates in ASD may be related to the disruption in the TCA cycle. As less ATP is produced through the TCA cycle, an increase in the glycolytic pathway may be needed to meet the ATP demand of the cell. The anomaly in the function of the TCA cycle can also be deducted from the ratio of CO_2_ secreted for every glucose molecule uptook. Theoretically, 6 CO_2_ molecules are formed per glucose molecule by complete aerobic respiration, while none are formed after glycolysis. As seen in Figure 4, the CO_2_ secretion per glucose uptake for the ASD-specific model is found to be lower compared to the control model with a large effect size, which is an indicator of incomplete aerobic respiration.

The disturbance of the TCA cycle reduces the aerobic respiration rate, and this phenomenon generally results in elevated levels of pyruvate, as well as its derivatives, lactate and alanine [16]. In our metabolic brain model, the secretion of pyruvate is found to be significantly greater in the ASD model and lactate is found to be generally transferred into the cell, yet the ASD model needed less lactate than the control model. Pyruvate is converted to alanine by alanine aminotransferase (ALT), whereas the pyruvate-derivative oxaloacetate is converted to aspartate by the aspartate aminotransferase (AST) enzyme. Our simulations show that pyruvate secretion and the activity of reactions catalyzed by ALT and AST are higher in the ASD model, thus supporting the reduced aerobic respiration hypothesis (Figure 4). Several studies also revealed elevated ALT and AST levels in the bodily fluids of ASD patients [51], confirming our results. The disturbances in the TCA cycle may also be explained by the elevated levels of ammonia in the cell, which is a byproduct of protein metabolism and is accepted as another biomarker of the mitochondrial disorder [52,53,54]. Our results showed that the ASD model secretes higher ammonia on average (Figure 4). Elevated ammonia levels may constrain the TCA cycle in neurons and glia by inhibiting cycle enzymes such as pyruvate dehydrogenase (PDH), isocitrate dehydrogenase, (IDH), and α-ketoglutarate dehydrogenase (α-KGDH).

### 3.3. ASD-Related Changes in Mitochondrial Dysfunction and Oxidative Stress Related Markers

**Figure 5:**
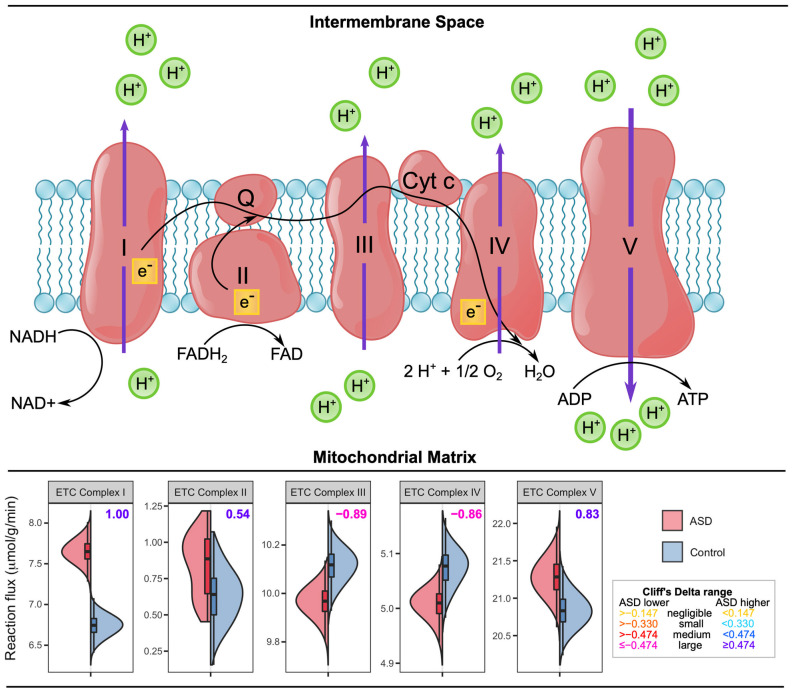
Reaction fluxes for electron transport chain complexes in µmol/(g min) acquired by uniform sampling of the ASD-specific and control models. The numbers of the upper right corner of each graph denote the Cliff’s d values.

The mitochondrial electron transport chain (ETC) is located in the inner membrane of the mitochondria and produces ATP by generating a proton gradient between the mitochondrial matrix to the inner membrane space. ETC consists of 5 complexes: complex I (NADH dehydrogenase), complex II (succinate dehydrogenase), complex III (cytochrome bc1 complex), complex IV (cytochrome c oxidase), and complex V (ATP synthase) (Figure 5). We computationally observed some unusual results (Figure 5). Among five complexes, ETC complexes I and V show higher flux values in the ASD model, while the remaining complexes have lower fluxes, with all complexes showing large Cliff’s d values. The ETC complex protein levels were measured in the cerebellum, frontal, parietal, occipital, and temporal cortices of ASD patients and controls of different ages, and these ETC complex levels were found to be lower in the cerebellum, frontal cortex, and temporal cortex of the ASD cases aged between 4 and 10 compared to the age-matched controls, but not in other cortices and ASD cases aged between 14 and39 [55]. Specifically, the levels of complex I are significantly decreased in the frontal cortex of autistic children, with non-significant decreases in levels of other complexes. Another study by the same group compared the ETC activities in the frontal cortex of ASD patients and detected that 9 out of 14 ASD patients had at least one ETC complex with lower activity than controls. The most affected complexes were found as complexes I and V [56]. The decrease in the complex I activity in blood cells was also been found in recent studies [51]. The activities for all the ETC complexes in the ASD granulocytes were also significantly lower [57]. Another late study also detected abnormal complex activities in the ASD fibroblasts [58]. Although our simulation results on the ETC complex (Figure 5) were unanticipated, none of the aforementioned activity studies were conducted on brain tissues, and ETC complex functions can vary in different tissues and even in the same tissue.

Oxidative stress is believed to be a chronic condition in ASD as no studies have found a correlation between age and the levels of oxidative stress markers in ASD patients [59]. Glutathione peroxidase (GPx) is a significant antioxidant enzyme that takes part in the peroxyl scavenging mechanism by converting GSH and hydrogen peroxide (H_2_O_2_) to GSSG and water. Our results reveal that the total rate of GPx-catalyzed reactions in the cytosol and mitochondria have similar flux ranges in both models, indicating that both the ASD and control models scavenge similar amounts of H_2_O_2_ (Figure 4). In the literature, the GPx activities in the erythrocytes and plasma of ASD patients were found to be low [60,61], as well as in the cerebellum [62], with two conflicting results [63,64] suggesting there is not a pattern regarding GPx in ASD. The reaction converting GSSG to GSH is catalyzed by glutathione-disulfide reductase (GSR), which is considered to play a significant part in antioxidant production in cells. Our simulation results show that the fluxes for the reaction catalyzed by GSR are significantly increased in ASD cases, suggesting inflated oxidative stress in the prefrontal cortex of ASD individuals. However, the role of GSR in ASD is still poorly understood, with previous studies finding conflicting results. One study detected no significant difference between the ASD and control groups, yet 60% of the ASD cases showed lower and higher GSR activities than 95% CI (confidence interval) of the control group [62]. The other study detected higher GSR activity in the plasma of ASD patients [64].

Superoxide dismutase (SOD) and catalase (CAT) are two other significant antioxidant enzymes. SOD catalyzes the dismutation of superoxide anion ($O_{2}^{-}$) to H_2_O_2_, whereas CAT catalyzes the conversion of H_2_O_2_ to H_2_O. Our simulation result for the SOD enzyme is displayed in Figure 4. The mean of the reaction flux is lower in the ASD model, but the range for the flux is wider than that in the control model, which suggests that the ASD model has a higher variability for SOD activity compared to the control model in accordance with several literature findings. In fact, literature studies have presented conflicting results. Lower [61], higher [65,66,67], or similar [63,68] SOD activities in ASD plasma and erythrocytes have been reported in comparison to controls. The same trend was also detected for CAT: lower [66,67], higher [65,69], and non-significant difference [68]. The mean values of CAT activities are closer in our ASD and control models, with a slight elevation in ASD, yet the maximum CAT activities are greater in the ASD model. The overactivity of oxidative stress-protective enzymes such as CAT, GSR, and SOD in our ASD model may indicate that these enzymes should overwork to compensate for the oxidative stress in the ASD brain.

### 3.4. Neuroinflammatory Markers in ASD

Neuroinflammation is the inflammation of the central nervous system and is indicated to take part in the pathogenesis of several neurological diseases. The activation of glial cells, such as astrocytes, which are one of the most abundant cells in the prefrontal cortex, is associated with the neuroinflammation status of the brain and releases pro-inflammatory mediators. The inflammatory status of ASD children has been indicated by the increase in the pro-inflammatory cytokines [12]. In this section, we present our computational findings on exchange fluxes of neuroinflammation-related compounds and also elaborate on the metabolite biomarkers for inflammation.

**Figure 6:**
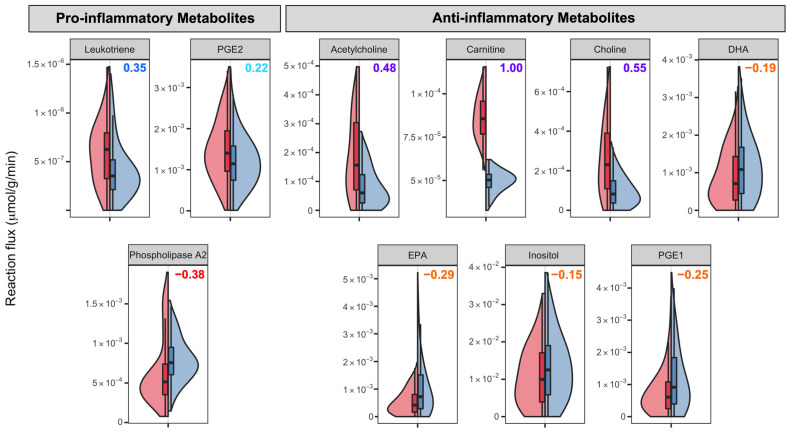
Reaction fluxes for several inflammatory markers in µmol/(g min) acquired by uniform sampling of the ASD-specific and control models. The numbers in the upper right corner of each graph denote the Cliff’s d values. The color codes for Cliff’s d values can be seen in Figure 5. DHA: Docosahexaenoic acid, ENA: Eicosapentaenoic acid, PGE1: Prostaglandin E1, PGE2: Prostaglandin E2.

Inositol (also known as myo-inositol) and choline-containing compounds are two indicators of neuroinflammation in the brain and are generally detected via proton magnetic resonance spectroscopy analyses. Activation of glial cells is thought to be correlated with higher inositol and choline-containing compound levels [70]. The activation in these cells is also associated with the neuroinflammation status of the brain and the release of pro-inflammatory mediators [71], suggesting that inositol and choline levels may be relevant to the neuroinflammation status. Our simulation results are shown in Figure 6. Acetylcholine and choline secretions are found to be higher in the ASD model with large effect sizes. Although the difference is not significant for inositol, the control model secretes more inositol on average. We may speculate that although the literature suggests no significant difference for these metabolites in prefrontal cortex [72], the neuroinflammation in the prefrontal cortex stimulates the astrocyte activation and its relevant reactions, which in turn produces more choline-related metabolites.

Phospholipases, enzymes hydrolyzing phospholipids to fatty acids, are at the center of the fatty acid mechanism. Specifically, phospholipase A2 (PLA2) breaks the phospholipids into arachidonic acid and polyunsaturated free fatty acids (PUFA), which are further hydrolyzed to eicosanoids such as prostaglandins and leukotrienes. We found that the reaction catalyzed by the PLA2 enzyme has significantly higher fluxes in the control model (Figure 6). A small number of studies investigated the activity of PLA2 in ASD and found conflicting results; in particular, one study detected lower PLA2 activity in the serum of ASD children [73], and two others concluded higher activities in the RBC [74] and blood [75] of ASD children. Docosahexaenoic acid (DHA) is the most abundant n-3 PUFA in the brain and regulates the function of glial cells and neurons. It also protects the neurons from damage in several brain diseases and diminishes neuroinflammation [76,77]. The precursor of DHA, eicosapentaenoic acid (EPA), is also effective against inflammation, although it is less abundant than DHA in the brain [78]. Our simulations show that both DHA and EPA secretions are higher in the control model, although the difference is not very explicit, indicating that ASD people may be more prone to inflammation and cell damage (Figure 6). Studies have shown that the DHA levels are lower in the blood of ASD children, whereas only one study reported significantly lower levels of EPA in the ASD brain [78]. The differences in these fatty acids’ secretion capacities indeed indicate perturbations in fatty acid metabolism.

Prostaglandins and leukotrienes are two important classes of eicosanoids present in inflammatory conditions. Prostaglandin E2 (PGE2) is one of the most abundant prostaglandins in the human body and is a mediator for many biological functions, including inflammation [79]. PGE2 may induce both pro-inflammatory and anti-inflammatory processes depending on the context, but mostly in the pro-inflammatory direction [80]. On the other hand, prostaglandin E1 (PGE1) is thought to function as an anti-inflammatory mediator for several animals, including humans [81,82]. According to our simulations, the secretion of PGE2 from the cell, often a pro-inflammatory metabolite, is higher in the ASD model, whereas the PGE1 secretion is lower. Leukotrienes, especially leukotriene B4, are indicated as pro-inflammatory mediators and suggested as potential therapeutic targets for the modulation of inflammation [83]. The leukotriene levels were found to be higher in the ASD model compared to those in neurotypical controls (Figure 6). These results, combined with the literature findings [84,85,86,87] on the bodily fluids of ASD patients, imply that there is inflammatory stress in the prefrontal cortex of ASD individuals.

### 3.5. Sphingolipid Metabolism Changes in ASD

Compared to other tissues, the nervous system, especially the brain, has the highest lipid content and complexity. Sphingolipids, being one of the major classes of lipids that are essential for eukaryotic cells, are highly enriched in the brain. They have several structural roles in the plasma membranes and are also signaling molecules regulating cellular events such as cell growth, differentiation, senescence, and apoptosis [88,89]. Sphingolipids are implicated as a critical component for brain development and function. Altered sphingolipid metabolism has been implied in several neurological and psychiatric disorders. Thus far, no detailed studies have focused on the altered metabolism of sphingolipids in the brains of ASD patients and its effects on the pathophysiology of the disease. Currently, only two sphingolipid metabolites have been reported in the literature for ASD. Sphingosine-1-phosphate (S1P) was found to be significantly increased in ASD serum [90], and several ceramide types have been elevated in the ASD brain [91] and prefrontal cortex [92].

**Figure 7:**
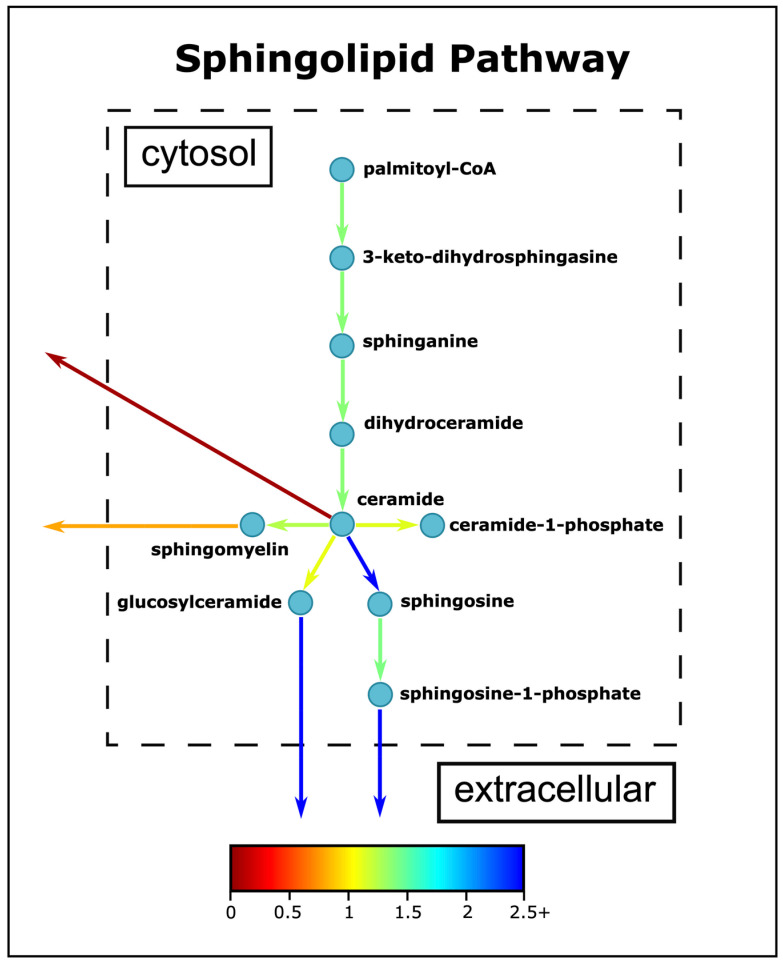
Simplified sphingolipid pathway. The colors indicate the ratio of average fluxes of ASD to control model.

Ceramides participate in important signaling processes, such as inflammation, and can be generated by pro-inflammatory cytokines [93]. Ceramide can be phosphorylated to ceramide-1-phosphate, which may activate PLA2, and may thus induce inflammation. Ceramides can also be degraded to sphingosines, which can be converted to S1P. S1P has many roles in the cell, including cell migration, proliferation, cellular architecture, and inflammation [94], and is known to have anti-inflammatory effects [95]. Our simulations have detected several differences in the sphingolipid metabolisms of ASD and neurotypical people (Figure 7). On average, the sphingolipid metabolism has higher fluxes in the ASD model, but the ratios through the pathway are consistent with each other, except for ceramide conversion to sphingosine. The fluxes through the pathway are nearly 1.2 to 1.4 times faster in ASD, except for the conversion of ceramide to sphingosine. The S1P secretion fluxes are increased in our simulations (Figure 7), in parallel with the literature results [90], while the pro-inflammatory ceramide secretion is restrained in ASD individuals on average, contrary to our expectations. Although more ceramide is produced in the ASD model, most of the produced ceramide is converted to other derivatives like sphingomyelin or sphingosine, which may explain the decrease in ceramide secretion. The increase in ceramide production can be induced by oxidative stress [96], which is shown to be elevated in ASD children by this study. Another derivative of ceramides, glucosylceramides, are indicated as both pro- and anti-inflammatory mediators and are also found to be higher in ASD patients.

On a special note, our simulations indicated altered sphingolipid metabolism between the ASD and neurotypical control models and this metabolism needs further research to shed light on the effect of sphingolipids on the pathogenesis of ASD. The research on the relationship between sphingolipid metabolism and ASD has been limited. In fact, the majority of studies on ASD omics have focused on transcriptomics or metabolomics, and it is only recently that lipidomics analyses have been conducted, although lipids constitute 36–66% of the dry weight of the human brain depending on grey or white matter, and nearly one-third of the total lipids are sphingolipids [97]. The increase in the number of lipidomics studies may enhance our understanding of the brain structure and function in ASD children.

## 4. Conclusions

Our simulations on reconstructed ASD-specific and neurotypical control GEMs demonstrated that several pathways, which have already been shown to be disturbed in ASD, generally yield complementary results with literature findings. The energy metabolism is altered in ASD, as indicated by the distinct patterns in glycolysis and the TCA cycle. One of the most prevalent comorbidities of ASD, mitochondrial dysfunction, is also supported by the disparities in the activities of ETC complexes and the increase in the mitochondrial dysfunction biomarkers. In addition, the reactions catalyzed by the oxidative stress marker enzymes, as well as the ones catalyzed by oxidative-stress protective enzymes, showed higher fluxes in ASD, indicating the presence of oxidative stress in ASD. Pro-inflammatory markers are found to have higher fluxes in ASD, whereas contradictory results are found for anti-inflammatory metabolites. Sphingolipid metabolites, especially S1P and ceramide, are promising biomarkers for ASD and should be thoroughly investigated by in vitro experiments.

Omics data, such as transcriptomics, proteomics, and metabolomics, enhance the predictive power of systems biology tools such as genome-scale modeling. However, the lack of adequate knowledge about ASD was the most significant challenge encountered throughout this modeling study. Despite the limited number of omics studies conducted on the brains of ASD patients, all of them are post-mortem studies due to inaccessibility to the area, which may have distinctive features compared to ante-mortem analyses. Additionally, the number of studies reporting the enzyme activities in the brain, specifically the prefrontal cortex, is quite low, and in most cases, they conflict with each other. Thus, the neuro-modeling studies, including ours, have inadequate resources to validate the computational results. Furthermore, the heterogeneity of the disease and the complexity of the nervous system hinders the efforts to understand the underlying conditions behind ASD and the impact of altered pathways on the symptoms of ASD. However, customized forecasts of patients’ phenotypic characterization can be improved by employing genome-scale metabolic models. The metabolic models tailored to individual conditions, tissues, and patients shed light on cell-specific changes in the metabolic network, allowing for the prediction of disease metabolism biomarkers. All in all, we may conclude that genome-scale modeling may be beneficial in investigating the disturbed mechanisms in ASD.

## Figures and Tables

**Table 1:** ASD-specific metabolic traits predicted by the genome scale metabolic brain model.

ASD-Specific Markers Found in		Pathways/Metabolites	Main Findings (ASD/Control)
**Energy Metabolism**		Glycolysis	↑
		TCA cycle	↓
		ETC I, II, V	↑
		ETC III, IV	↓
**Oxidative Stress**		Ammonia, lactate, pyruvate	↑
		CO_2_/Glucose	↓
**Mitochondrial Dysfunction**		ALT, AST, GSR	↑
		CS, PDH	↓
**Neuroinflammation**	Pro-inflammatory	Leukotriene, PGE2	↑
		PLA2	↓
	Anti-inflammatory	Carnitine, choline	↑
		DHA, EPA, inositol, PGE1	↓
**Sphingolipid Metabolism**		Glucosylceramide, S1P	↑
		Ceramide	↓

## Data Availability

Publicly available datasets were analyzed in this study. The constructed models for this study are available at https://doi.org/10.5281/zenodo.7602974.

## Supplementary Materials

The following supporting information can be downloaded at: https://www.mdpi.com/article/10.3390/biomedicines11020583/s1, Figure S1: The distribution of the median quantile normalized gene expression values for samples aged between 2 and 14.

## References

[1] Lord C., Elsabbagh M., Baird G., Veenstra-Vanderweele J. (2018). Autism Spectrum Disorder. Lancet.

[2] Zeidan J., Fombonne E., Scorah J., Ibrahim A., Durkin M.S., Saxena S., Yusuf A., Shih A., Elsabbagh M. (2022). Global Prevalence of Autism: A Systematic Review Update. Autism Res..

[3] Amaral D.G., Schumann C.M., Nordahl C.W. (2008). Neuroanatomy of Autism. Trends Neurosci..

[4] Van Rooij D., Anagnostou E., Arango C., Auzias G., Behrmann M., Busatto G.F., Calderoni S., Daly E., Deruelle C., Di Martino A. (2018). Cortical and Subcortical Brain Morphometry Differences between Patients with Autism Spectrum Disorder and Healthy Individuals across the Lifespan: Results from the ENIGMA ASD Working Group. Am. J. Psychiatry.

[5] Carlisi C.O., Norman L.J., Lukito S.S., Radua J., Mataix-Cols D., Rubia K. (2017). Comparative Multimodal Meta-Analysis of Structural and Functional Brain Abnormalities in Autism Spectrum Disorder and Obsessive-Compulsive Disorder. Biol. Psychiatry.

[6] Courchesne E., Pierce K. (2005). Why the Frontal Cortex in Autism Might Be Talking Only to Itself: Local over-Connectivity but Long-Distance Disconnection. Curr. Opin. Neurobiol..

[7] Courchesne E., Mouton P.R., Calhoun M.E., Semendeferi K., Ahrens-Barbeau C., Hallet M.J., Barnes C.C., Pierce K. (2011). Neuron Number and Size in Prefrontal Cortex of Children With Autism. JAMA.

[8] Morgan J.T., Chana G., Pardo C.A., Achim C., Semendeferi K., Buckwalter J., Courchesne E., Everall I.P. (2010). Microglial Activation and Increased Microglial Density Observed in the Dorsolateral Prefrontal Cortex in Autism. Biol. Psychiatry.

[9] Abraham J.R., Szoko N., Barnard J., Rubin R.A., Schlatzer D., Lundberg K., Li X., Natowicz M.R. (2019). Proteomic Investigations of Autism Brain Identify Known and Novel Pathogenetic Processes. Sci. Rep..

[10] Park D.I. (2020). Genomics, Transcriptomics, Proteomics and Big Data Analysis in the Discovery of New Diagnostic Markers and Targets for Therapy Development.

[11] Rossignol D.A., Frye R.E. (2012). A Review of Research Trends in Physiological Abnormalities in Autism Spectrum Disorders: Immune Dysregulation, Inflammation, Oxidative Stress, Mitochondrial Dysfunction and Environmental Toxicant Exposures. Mol. Psychiatry.

[12] Siniscalco D., Schultz S., Brigida A., Antonucci N. (2018). Inflammation and Neuro-Immune Dysregulations in Autism Spectrum Disorders. Pharmaceuticals.

[13] Ford T.C., Crewther D.P. (2016). A Comprehensive Review of the 1H-MRS Metabolite Spectrum in Autism Spectrum Disorder. Front. Mol. Neurosci..

[14] Likhitweerawong N., Thonusin C., Boonchooduang N., Louthrenoo O., Nookaew I., Chattipakorn N., Chattipakorn S.C. (2021). Profiles of Urine and Blood Metabolomics in Autism Spectrum Disorders. Metab. Brain Dis..

[15] Bjørklund G., Meguid N.A., El-Bana M.A., Tinkov A.A., Saad K., Dadar M., Hemimi M., Skalny A.V., Hosnedlová B., Kizek R. (2020). Oxidative Stress in Autism Spectrum Disorder. Mol. Neurobiol..

[16] Rossignol D.A., Frye R.E. (2012). Mitochondrial Dysfunction in Autism Spectrum Disorders: A Systematic Review and Meta-Analysis. Mol. Psychiatry.

[17] Gu C., Kim G.B., Kim W.J., Kim H.U., Lee S.Y. (2019). Current Status and Applications of Genome-Scale Metabolic Models. Genome Biol..

[18] Lewis J.E., Kemp M.L. (2021). Integration of Machine Learning and Genome-Scale Metabolic Modeling Identifies Multi-Omics Biomarkers for Radiation Resistance. Nat. Commun..

[19] Moolamalla S.T.R., Vinod P.K. (2020). Genome-Scale Metabolic Modelling Predicts Biomarkers and Therapeutic Targets for Neuropsychiatric Disorders. Comput. Biol. Med..

[20] Chow M.L., Li H.-R., Winn M.E., April C., Barnes C.C., Wynshaw-Boris A., Fan J.-B., Fu X.-D., Courchesne E., Schork N.J. (2011). Genome-Wide Expression Assay Comparison across Frozen and Fixed Postmortem Brain Tissue Samples. BMC Genom..

[21] Chow M.L., Winn M.E., Li H.-R., April C., Wynshaw-Boris A., Fan J.-B., Fu X.-D., Courchesne E., Schork N.J. (2012). Preprocessing and Quality Control Strategies for Illumina DASL Assay-Based Brain Gene Expression Studies with Semi-Degraded Samples. Front. Genet..

[22] Chow M.L., Pramparo T., Winn M.E., Barnes C.C., Li H.-R., Weiss L., Fan J.-B., Murray S., April C., Belinson H. (2012). Age-Dependent Brain Gene Expression and Copy Number Anomalies in Autism Suggest Distinct Pathological Processes at Young Versus Mature Ages. PLoS Genet..

[23] Agren R., Mardinoglu A., Asplund A., Kampf C., Uhlen M., Nielsen J. (2014). Identification of Anticancer Drugs for Hepatocellular Carcinoma through Personalized Genome-scale Metabolic Modeling. Mol. Syst. Biol..

[24] Robinson J.L., Kocabaş P., Wang H., Cholley P.-E., Cook D., Nilsson A., Anton M., Ferreira R., Domenzain I., Billa V. (2020). An Atlas of Human Metabolism. Sci. Signal..

[25] Mardinoglu A., Agren R., Kampf C., Asplund A., Uhlen M., Nielsen J. (2014). Genome-Scale Metabolic Modelling of Hepatocytes Reveals Serine Deficiency in Patients with Non-Alcoholic Fatty Liver Disease. Nat. Commun..

[26] Blais E.M., Rawls K.D., Dougherty B.V., Li Z.I., Kolling G.L., Ye P., Wallqvist A., Papin J.A. (2017). Reconciled Rat and Human Metabolic Networks for Comparative Toxicogenomics and Biomarker Predictions. Nat. Commun..

[27] Brunk E., Sahoo S., Zielinski D.C., Altunkaya A., Dräger A., Mih N., Gatto F., Nilsson A., Preciat Gonzalez G.A., Aurich M.K. (2018). Recon3D Enables a Three-Dimensional View of Gene Variation in Human Metabolism. Nat. Biotechnol..

[28] Wang H., Marcišauskas S., Sánchez B.J., Domenzain I., Hermansson D., Agren R., Nielsen J., Kerkhoven E.J. (2018). RAVEN 2.0: A Versatile Toolbox for Metabolic Network Reconstruction and a Case Study on Streptomyces Coelicolor. PLoS Comput. Biol..

[29] Heirendt L., Arreckx S., Pfau T., Mendoza S.N., Richelle A., Heinken A., Haraldsdóttir H.S., Wachowiak J., Keating S.M., Vlasov V. (2019). Creation and Analysis of Biochemical Constraint-Based Models Using the COBRA Toolbox v.3.0. Nat. Protoc..

[30] Gurobi Optimization, LLC (2022). Gurobi Optimizer Reference Manual: Houston, Texas, USA. https://www.gurobi.com/wp-content/plugins/hd_documentations/documentation/9.0/refman.pdf.

[31] De Volder A., Bol A., Michel C., Congneau M., Goffinet A.M. (1987). Brain Glucose Metabolism in Children with the Autistic Syndrome: Positron Tomography Analysis. Brain Dev..

[32] Herold S., Frackowiak R.S.J., Le Couteur A., Rutter M., Howlin P. (1988). Cerebral Blood Flow and Metabolism of Oxygen and Glucose in Young Autistic Adults. Psychol. Med..

[33] Thorngren-Jerneck K., Ohlsson T., Sandell A., Erlandsson K., Strand S.E., Ryding E., Svenningsen N.W. (2001). Cerebral Glucose Metabolism Measured by Positron Emission Tomography in Term Newborn Infants with Hypoxic Ischemic Encephalopathy. Pediatr. Res..

[34] Attwell D., Laughlin S.B. (2001). An Energy Budget for Signaling in the Grey Matter of the Brain. J. Cereb. Blood Flow Metab..

[35] Hogstad S., Svenneby G., Torgner I.A., Kvamme E., Hertz L., Schousboe A. (1988). Glutaminase in Neurons and Astrocytes Cultured from Mouse Brain: Kinetic Properties and Effects of Phosphate, Glutamate, and Ammonia. Neurochem. Res..

[36] Jeitner T.M., Cooper A.J.L. (2014). Inhibition of Human Glutamine Synthetase by L-Methionine-S,R-Sulfoximine—Relevance to the Treatment of Neurological Diseases. Metab. Brain Dis..

[37] Ureña-Guerrero M.E., López-Pérez S.J., Beas-Zárate C. (2003). Neonatal Monosodium Glutamate Treatment Modifies Glutamic Acid Decarboxylase Activity during Rat Brain Postnatal Development. Neurochem. Int..

[38] Perry T.L., Hansen S., Christie R.G. (1978). Amino Compounds and Organic Acids in CSF, Plasma, and Urine of Autistic Children. Biol. Psychiatry.

[39] (2016). Torchiano, Marco Effsize—A Package for Efficient Effect Size Computation. https://zenodo.org/record/196082#.Y-3ThnYzZPY.

[40] Hladky S.B., Barrand M.A. (2014). Mechanisms of Fluid Movement into, through and out of the Brain: Evaluation of the Evidence. Fluids Barriers CNS.

[41] Bak L.K., Schousboe A., Waagepetersen H.S. (2006). The Glutamate/GABA-Glutamine Cycle: Aspects of Transport, Neurotransmitter Homeostasis and Ammonia Transfer. J. Neurochem..

[42] Rose S., Niyazov D.M., Rossignol D.A., Goldenthal M., Kahler S.G., Frye R.E. (2018). Clinical and Molecular Characteristics of Mitochondrial Dysfunction in Autism Spectrum Disorder. Mol. Diagnosis Ther..

[43] He Y., Zhou Y., Ma W., Wang J. (2019). An Integrated Transcriptomic Analysis of Autism Spectrum Disorder. Sci. Rep..

[44] Ginsberg M.R., Rubin R.A., Falcone T., Ting A.H., Natowicz M.R. (2012). Brain Transcriptional and Epigenetic Associations with Autism. PLoS ONE.

[45] Rose S., Bennuri S.C., Wynne R., Melnyk S., James S.J., Frye R.E. (2017). Mitochondrial and Redox Abnormalities in Autism Lymphoblastoid Cells: A Sibling Control Study. FASEB J..

[46] Denton R.M. (2009). Regulation of Mitochondrial Dehydrogenases by Calcium Ions. Biochim. Biophys. Acta Bioenerg..

[47] Liao X., Li Y. (2020). Genetic Associations between Voltage-Gated Calcium Channels and Autism Spectrum Disorder: A Systematic Review. Mol. Brain.

[48] Yehia L., Ni Y., Feng F., Seyfi M., Sadler T., Frazier T.W., Eng C. (2019). Distinct Alterations in Tricarboxylic Acid Cycle Metabolites Associate with Cancer and Autism Phenotypes in Cowden Syndrome and Bannayan-Riley-Ruvalcaba Syndrome. Am. J. Hum. Genet..

[49] Orozco J.S., Hertz-Picciotto I., Abbeduto L., Slupsky C.M. (2019). Metabolomics Analysis of Children with Autism, Idiopathic-Developmental Delays, and Down Syndrome. Transl. Psychiatry.

[50] Rangel-Huerta O.D., Gomez-Fernández A., de la Torre-Aguilar M.J., Gil A., Perez-Navero J.L., Flores-Rojas K., Martín-Borreguero P., Gil-Campos M. (2019). Metabolic Profiling in Children with Autism Spectrum Disorder with and without Mental Regression: Preliminary Results from a Cross-Sectional Case–Control Study. Metabolomics.

[51] Mahalaxmi I., Subramaniam M.D., Gopalakrishnan A.V., Vellingiri B. (2021). Dysfunction in Mitochondrial Electron Transport Chain Complex I, Pyruvate Dehydrogenase Activity, and Mutations in ND1 and ND4 Gene in Autism Spectrum Disorder Subjects from Tamil Nadu Population, India. Mol. Neurobiol..

[52] Filipek P.A., Juranek J., Nguyen M.T., Cummings C., Gargus J.J. (2004). Relative Carnitine Deficiency in Autism. J. Autism Dev. Disord..

[53] Saleem T.H., Shehata G.A., Toghan R., Sakhr H.M., Bakri A.H., Desoky T., Hamdan F.R.A., Mohamed N.F., Hassan M.H. (2020). Assessments of Amino Acids, Ammonia and Oxidative Stress Among Cohort of Egyptian Autistic Children: Correlations with Electroencephalogram and Disease Severity [Corrigendum]. Neuropsychiatr. Dis. Treat..

[54] Shahjadi S., Khan A.S., Ahmed M.U. (2017). Mitochondrial Dysfunction in Early Diagnosed Autism Spectrum Disorder Children. J. Dhaka Med. Coll..

[55] Chauhan A., Gu F., Essa M.M., Wegiel J., Kaur K., Brown W.T., Chauhan V. (2011). Brain Region-Specific Deficit in Mitochondrial Electron Transport Chain Complexes in Children with Autism. J. Neurochem..

[56] Gu F., Chauhan V., Kaur K., Brown W.T., LaFauci G., Wegiel J., Chauhan A. (2013). Alterations in Mitochondrial DNA Copy Number and the Activities of Electron Transport Chain Complexes and Pyruvate Dehydrogenase in the Frontal Cortex from Subjects with Autism. Transl. Psychiatry.

[57] Napoli E., Wong S., Hertz-Picciotto I., Giulivi C. (2014). Deficits in Bioenergetics and Impaired Immune Response in Granulocytes From Children With Autism. Pediatrics.

[58] Frye R.E., Lionnard L., Singh I., Karim M.A., Chajra H., Frechet M., Kissa K., Racine V., Ammanamanchi A., McCarty P.J. (2021). Mitochondrial Morphology Is Associated with Respiratory Chain Uncoupling in Autism Spectrum Disorder. Transl. Psychiatry.

[59] Rose S., Melnyk S., Pavliv O., Bai S., Nick T.G., Frye R.E., James S.J. (2012). Evidence of Oxidative Damage and Inflammation Associated with Low Glutathione Redox Status in the Autism Brain. Transl. Psychiatry.

[60] Meguid N.A., Dardir A.A., Abdel-Raouf E.R., Hashish A. (2011). Evaluation of Oxidative Stress in Autism: Defective Antioxidant Enzymes and Increased Lipid Peroxidation. Biol. Trace Elem. Res..

[61] Yorbik O., Sayal A., Akay C., Akbiyik D.I., Sohmen T. (2002). Investigation of Antioxidant Enzymes in Children with Autistic Disorder. Prostaglandins Leukot. Essent. Fat. Acids.

[62] Gu F., Chauhan V., Chauhan A. (2013). Impaired Synthesis and Antioxidant Defense of Glutathione in the Cerebellum of Autistic Subjects: Alterations in the Activities and Protein Expression of Glutathione-Related Enzymes. Free Radic. Biol. Med..

[63] Söğüt S., Zoroğlu S.S., Özyurt H., Ramazan Yılmaz H., Özuğurlu F., Sivaslı E., Yetkin Ö., Yanık M., Tutkun H., Savaş H.A. (2003). Changes in Nitric Oxide Levels and Antioxidant Enzyme Activities May Have a Role in the Pathophysiological Mechanisms Involved in Autism. Clin. Chim. Acta.

[64] Kondolot M., Ozmert E.N., Ascı A., Erkekoglu P., Oztop D.B., Gumus H., Kocer-Gumusel B., Yurdakok K. (2016). Plasma Phthalate and Bisphenol a Levels and Oxidant-Antioxidant Status in Autistic Children. Environ. Toxicol. Pharmacol..

[65] Altun H., Şahin N., Kurutaş E.B., Karaaslan U., Sevgen F.H., Fındıklı E. (2018). Assessment of Malondialdehyde Levels, Superoxide Dismutase, and Catalase Activity in Children with Autism Spectrum Disorders. Psychiatry Clin. Psychopharmacol..

[66] Al-Gadani Y., El-Ansary A., Attas O., Al-Ayadhi L. (2009). Metabolic Biomarkers Related to Oxidative Stress and Antioxidant Status in Saudi Autistic Children. Clin. Biochem..

[67] Zoroglu S.S., Armutcu F., Ozen S., Gurel A., Sivasli E., Yetkin O., Meram I. (2004). Increased Oxidative Stress and Altered Activities of Erythrocyte Free Radical Scavenging Enzymes in Autism. Eur. Arch. Psychiatry Clin. Neurosci..

[68] Ghezzo A., Visconti P., Abruzzo P.M., Bolotta A., Ferreri C., Gobbi G., Malisardi G., Manfredini S., Marini M., Nanetti L. (2013). Oxidative Stress and Erythrocyte Membrane Alterations in Children with Autism: Correlation with Clinical Features. PLoS ONE.

[69] Ranjbar A., Rashedi V., Rezaei M. (2014). Comparison of Urinary Oxidative Biomarkers in Iranian Children with Autism. Res. Dev. Disabil..

[70] Young A.M.H., Chakrabarti B., Roberts D., Lai M.-C., Suckling J., Baron-Cohen S. (2016). From Molecules to Neural Morphology: Understanding Neuroinflammation in Autism Spectrum Condition. Mol. Autism.

[71] Matta S.M., Hill-Yardin E.L., Crack P.J. (2019). The Influence of Neuroinflammation in Autism Spectrum Disorder. Brain. Behav. Immun..

[72] Shirayama Y., Matsumoto K., Osone F., Hara A., Guan S., Hamatani S., Muneoka K., Sato K., Okada A., Yokokawa T. (2022). The Lack of Alterations in Metabolites in the Medial Prefrontal Cortex and Amygdala, but Their Associations with Autistic Traits, Empathy, and Personality Traits in Adults with Autism Spectrum Disorder: A Preliminary Study. J. Autism Dev. Disord..

[73] Hayek J., Cervellati C., Crivellari I., Pecorelli A., Valacchi G. (2017). Lactonase Activity and Lipoprotein-Phospholipase A 2 as Possible Novel Serum Biomarkers for the Differential Diagnosis of Autism Spectrum Disorders and Rett Syndrome: Results from a Pilot Study. Oxid. Med. Cell. Longev..

[74] Bell J.G., MacKinlay E.E., Dick J.R., MacDonald D.J., Boyle R.M., Glen A.C.A. (2004). Essential Fatty Acids and Phospholipase A2 in Autistic Spectrum Disorders. Prostaglandins Leukot. Essent. Fat. Acids.

[75] Tostes M.H.F.d.S., Polonini H.C., Mendes R., Brandão M.A.F., Gattaz W.F., Raposo N.R.B. (2013). Fatty Acid and Phospholipase A2 Plasma Levels in Children with Autism. Trends Psychiatry Psychother..

[76] Fourrier C., Remus-Borel J., Greenhalgh A.D., Guichardant M., Bernoud-Hubac N., Lagarde M., Joffre C., Layé S. (2017). Docosahexaenoic Acid-Containing Choline Phospholipid Modulates LPS-Induced Neuroinflammation in Vivo and in Microglia in Vitro. J. Neuroinflamm..

[77] Orr S.K., Palumbo S., Bosetti F., Mount H.T., Kang J.X., Greenwood C.E., Ma D.W.L., Serhan C.N., Bazinet R.P. (2013). Unesterified Docosahexaenoic Acid Is Protective in Neuroinflammation. J. Neurochem..

[78] Tesei A., Crippa A., Ceccarelli S.B., Mauri M., Molteni M., Agostoni C., Nobile M. (2017). The Potential Relevance of Docosahexaenoic Acid and Eicosapentaenoic Acid to the Etiopathogenesis of Childhood Neuropsychiatric Disorders. Eur. Child Adolesc. Psychiatry.

[79] Ricciotti E., FitzGerald G.A. (2011). Prostaglandins and Inflammation. Arterioscler. Thromb. Vasc. Biol..

[80] Sreeramkumar V., Fresno M., Cuesta N. (2012). Prostaglandin E2 and T Cells: Friends or Foes?. Immunol. Cell Biol..

[81] Gezginci-Oktayoglu S., Orhan N., Bolkent S. (2016). Prostaglandin-E 1 Has a Protective Effect on Renal Ischemia/Reperfusion-Induced Oxidative Stress and Inflammation Mediated Gastric Damage in Rats. Int. Immunopharmacol..

[82] Fang W., Li H., Zhou L., Su L., Liang Y., Mu Y. (2010). Effect of Prostaglandin E1 on TNF-Induced Vascular Inflammation in Human Umbilical Vein Endothelial Cells. Can. J. Physiol. Pharmacol..

[83] Di Gennaro A., Haeggström J.Z. (2014). Targeting Leukotriene B4 in Inflammation. Expert Opin. Ther. Targets.

[84] Qasem H., Al-Ayadhi L., Bjørklund G., Chirumbolo S., El-Ansary A. (2018). Impaired Lipid Metabolism Markers to Assess the Risk of Neuroinflammation in Autism Spectrum Disorder. Metab. Brain Dis..

[85] El-Ansary A., Al-Ayadhi L. (2012). Lipid Mediators in Plasma of Autism Spectrum Disorders. Lipids Health Dis..

[86] Brigandi S., Shao H., Qian S., Shen Y., Wu B.-L., Kang J. (2015). Autistic Children Exhibit Decreased Levels of Essential Fatty Acids in Red Blood Cells. Int. J. Mol. Sci..

[87] Qasem H., Al-Ayadhi L., El-Ansary A. (2016). Cysteinyl Leukotriene Correlated with 8-Isoprostane Levels as Predictive Biomarkers for Sensory Dysfunction in Autism. Lipids Health Dis..

[88] Bartke N., Hannun Y.A. (2009). Bioactive Sphingolipids: Metabolism and Function. J. Lipid Res..

[89] Hannun Y.A., Obeid L.M. (2018). Sphingolipids and Their Metabolism in Physiology and Disease. Nat. Rev. Mol. Cell Biol..

[90] Wang H., Liang S., Wang M., Gao J., Sun C., Wang J., Xia W., Wu S., Sumner S.J., Zhang F. (2016). Potential Serum Biomarkers from a Metabolomics Study of Autism. J. Psychiatry Neurosci..

[91] Pardo C.A., Wheeler D., Vargas D.L., Haughey N.J., Zimmermann A. Abnormalities in Cholesterol, Ceramides and Markers of Oxidative Stress Are Revealed by Lipidomic Analysis of Brain Tissues in Autism. Proceedings of the International Meeting for Autism Research.

[92] Yu Q., He Z., Zubkov D., Huang S., Kurochkin I., Yang X., Halene T., Willmitzer L., Giavalisco P., Akbarian S. (2020). Lipidome Alterations in Human Prefrontal Cortex during Development, Aging, and Cognitive Disorders. Mol. Psychiatry.

[93] Arana L., Gangoiti P., Ouro A., Trueba M., Gómez-Muñoz A. (2010). Ceramide and Ceramide 1-Phosphate in Health and Disease. Lipids Health Dis..

[94] Snider A.J., Alexa Orr Gandy K., Obeid L.M. (2010). Sphingosine Kinase: Role in Regulation of Bioactive Sphingolipid Mediators in Inflammation. Biochimie.

[95] Hughes J.E., Srinivasan S., Lynch K.R., Proia R.L., Ferdek P., Hedrick C.C. (2008). Sphingosine-1-Phosphate Induces an Antiinflammatory Phenotype in Macrophages. Circ. Res..

[96] Brodowicz J., Przegaliński E., Müller C.P., Filip M. (2018). Ceramide and Its Related Neurochemical Networks as Targets for Some Brain Disorder Therapies. Neurotox. Res..

[97] O’Brien J.S., Sampson E.L. (1965). Lipid Composition of the Normal Human Brain: Gray Matter, White Matter, and Myelin. J. Lipid Res..

