# Supplementary material for: Neuroinflammation, Energy and Sphingolipid Metabolism Biomarkers Are Revealed by Metabolic Modeling of Autistic Brains
Source: Biomedicines. 2023 Feb 16;11(2):583. doi: 10.3390/biomedicines11020583 (PMC9953696; doi:10.3390/biomedicines11020583)

Supporting Information

Neuroinflammation, Energy and Sphingolipid Metabolism Biomarkers are Revealed by Metabolic Modeling of Autistic Brains

Elif Esvap, Kutlu O. Ulgen*

Department of Chemical Engineering, Bogazici University, Istanbul, Turkey

* Corresponding author. E-mail address: ulgenk@boun.edu.tr.

Figure S1. The distribution of the median quantile normalized gene expression values for samples aged between 2 and 14.

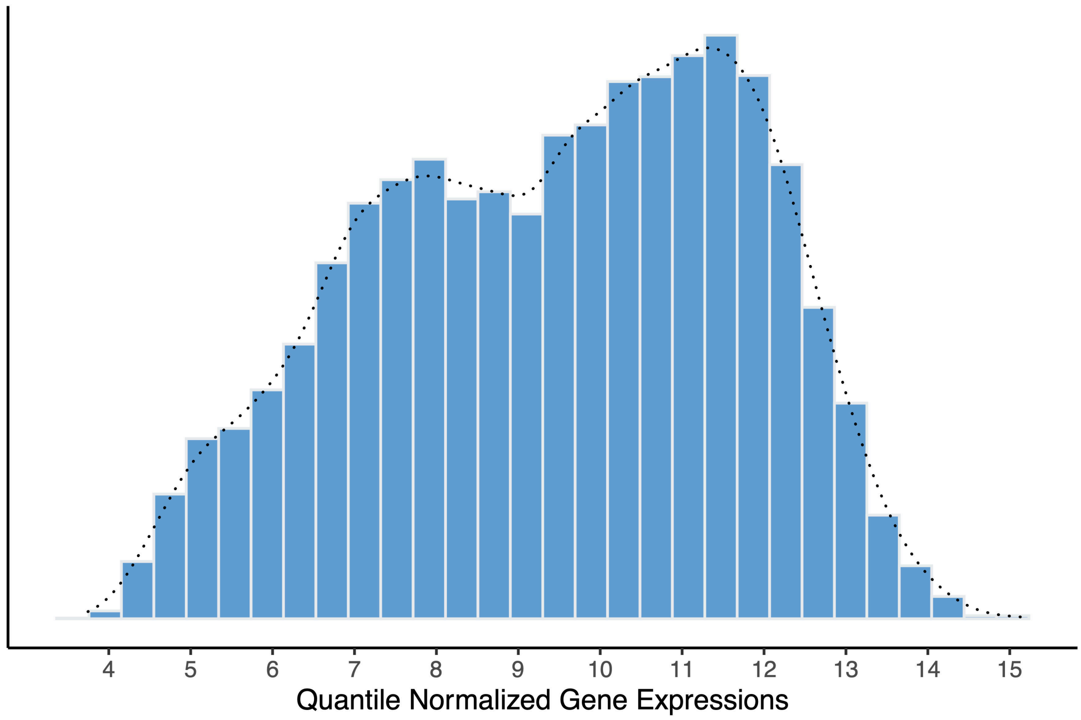

Supplement: Supplementary file 1 [file biomedicines-11-00583-s001.zip › SupportingInfo.pdf]
